# Development of learning objectives for neurology in a veterinary curriculum: Part II: Postgraduates

**DOI:** 10.1186/s12917-014-0314-4

**Published:** 2015-01-27

**Authors:** Yu-Wei Lin, Holger A Volk, Jacques Penderis, Thomas J Anderson, Sonia Añor, Alejandro Lujan-Feliu-Pascual, Veronika M Stein, Andrea Tipold, Jan P Ehlers

**Affiliations:** Department of Small Animal Medicine and Surgery, University of Veterinary Medicine Hannover, Buenteweg 9, Hannover, 30559 Germany; Department of Clinical Science and Service, Royal Veterinary College, University of London, London, UK; Department of Clinical Neurology, School of Veterinary Medicine, University of Glasgow, Glasgow, Scotland; Department of Animal Medicine and Surgery, The Autonomous University of Barcelona, Barcelona, Spain; Department of Animal Medicine and Surgery, CEU Cardenal Herrera University, Valencia, Spain; Didactics and Educational Research in Health Science, University Witten-Herdecke, Witten, Germany

**Keywords:** Veterinary education, Curriculum, Learning objectives, Neurology, Postgraduate, Diplomate, Resident, ECVN, ESVN, Europe

## Abstract

**Background:**

Specialization in veterinary medicine in Europe is organized through the Colleges of the European Board of Veterinary Specialization. To inform updating of the curriculum for residents of the European College of Veterinary Neurology (ECVN) job analysis was used. Defining job competencies of diploma holders in veterinary neurology can be used as references for curriculum design of resident training. With the support of the diplomates of the ECVN and the members of the European Society of Veterinary Neurology (ESVN) a mixed-method research, including a qualitative search of objectives and quantitative ranking with 149 Likert scale questions and 48 free text questions in 9 categories in a survey was conducted. In addition, opinions of different groups were subjected to statistical analysis and the result compared.

**Results:**

A return rate of 62% (n = 213/341) was achieved. Of the competencies identified by the Delphi process, 75% objectives were expected to attain expert level; 24% attain advanced level; 1% entry level. In addition, the exercise described the 11 highly ranked competencies, the 3 most frequently seen diseases of the central and peripheral nervous systems and the most frequently used immunosuppressive, antiepileptic and chemotherapeutic drugs.

**Conclusion:**

The outcomes of this “Delphi job analysis” provide a powerful tool to align the curriculum for ECVN resident training and can be adapted to the required job competencies, based on expectations. The expectation is that for majority of these competencies diplomates should attain an expert level. Besides knowledge and clinical skills, residents and diplomates are expected to demonstrate high standards in teaching and communication. The results of this study will help to create a European curriculum for postgraduate education in veterinary neurology.

**Electronic supplementary material:**

The online version of this article (doi:10.1186/s12917-014-0314-4) contains supplementary material, which is available to authorized users.

## Background

For the curricula of training programs for veterinary specialists to remain relevant the educational process must reflect the societies expectations of the professional attributes of veterinary specialists. The specialization in veterinary medicine was developed in the late 1980s; 26 veterinarians met at the World Small Animal Veterinary Association (WSAVA) congress in Harrogate, England in 1989 and discussed the specialization of veterinarians. Veterinary specialization is managed under the umbrella of the European Board of Veterinary Specialisation (EBVS), which was officially registered in 1996 [1,2]. The EBVS should recognize new specialty colleges and monitor their quality, register European veterinary specialists and promote specialist service in the public. In 1991, five European Colleges existed and the number increased to 23 in 2012 [3]. Individual colleges are responsible for their own training programs, with oversight from peer colleges within EBVS. In 2011 the Executive Committee of the European College of Veterinary Neurology (ECVN) instigated a process to inform curricular design through understanding the competencies expected of a diplomate.

The definition of competency has been developed to embrace societies expectations of professionals. Clavien, Nahrwold, Soper, & Bass [4], summarized the implications of competency as *“The business and industrial community recognizes that high-quality products and services are essential to compete in our global economy; Accordingly, the public has expectations that providers of services, including mechanics, hairdressers, lawyers and physicians, will be competent”*. The same principle can be adapted for the postgraduate education in veterinary neurology; one of the primary functions of the ECVN is to conduct examinations for veterinarians to get the ECVN Diploma and herewith certifying quality and competent services to the public and their animals [5]. Competency based process have been used for the design of human medical training programs from the 1990s [6,7] and in 2005, a European core curriculum for human neurology was presented [8] and a questionnaire-based survey on human neurology curricula was conducted from 2006 to 2009 to improve patient care in human neurology in Europe [9].

In contrast to human medicine no general core competencies in veterinary specialization training were developed. Instead, every college individually set up general learning objectives. Under the hypothesis of high standards of knowledge and skills reached by diplomates in veterinary neurology, the aim of this study was to create a catalogue of learning objectives for postgraduate training of veterinary neurology in Europe. By knowing the expectation of the job requirement of diplomate holders, the learning objectives for the resident training might be revealed.

A modified Delphi method (mixed-method-research) was conducted in the current study; two evaluation phases were applied for the collection of objective opinions regarding the competency domains expected of ECVN diplomats. The Delphi method has three important features: (1) anonymous response, (2) iteration with controlled feedback, (3) statistical group response (objective scores). These characteristics lower personal biases, group pressure [10] and minimize the “halo error” or “halo effect” [11] that makes the analysis of free expression of objective opinions, critics or judgments possible. Based on these characteristics, the opinions from experts could be collected and objective decisions could be justified.

The current manuscript obtains part II of the result of the whole survey. In the whole survey, the Participants were not only required to judge each learning objectives for job competencies of diplomates, but also for undergraduates training in pre-clinical and clinical year. The study of learning objectives veterinary neurology for undergraduates has been submitted in part I.

## Methods

The opinions of experts in the field of veterinary neurology were collected using a modified Delphi method, which consisted of qualitative and quantitative evaluation phases with subsequent statistical evaluation. The ECVN curriculum-working group initialized a draft list of competencies, which were revised and evaluated by members of the European Society of Veterinary Neurology (ESVN) and the ECVN.

### PHASE 1: Qualitative evaluation of competencies

The initial draft list of competencies was created by the ECVN curriculum working group consisting of seven selected veterinary neurologists* representing universities and private practices in Europe. The initial draft of competencies was based on the questionnaire created by the American College of Veterinary Internal Medicine (ACVIM) to assess competencies for their residents in neurology.

A draft list of competencies was formatted into 149 four-point Likert scale questions and 48 free text questions (Additional file 1) grouped into 9 categories:

1. Anatomy and Physiology
2. Pharmacology and Toxicology
3. Genetics and Molecular Biology
4. Clinical Methodology
5. Disease Mechanisms
6. Neuroanaesthesia and Neurosurgery
7. Neuroradiology
8. Pathology
9. Competencies in Academia

Moreover, for assessing the feedback 7 true-false questions and 1 free text question were developed to evaluate any potential differences between ECVN diplomates and advanced practitioners (Additional file 2). Eleven single and multiple-choice demographic questions were included to analyse the respondents.

### PHASE 2: Quantitative evaluations of competencies by ESVN and ECVN members

In the quantitative second phase the revised competencies were put into an online survey – (Surveymonkey®) with the login details distributed to 341 ESVN and ECVN members (142 ESVN members, 72 residents and 127 ECVN Diplomates). Residents are ESVN members. Since no statistical difference was found between residents and the other ESVN members, both groups were later combined in the statistical calculations.

Every participant received a unique link by e-mail, which was active for a 3 months period. During this period the users could pause and continue the questionnaire at any time. The curriculum development group estimated 1.5 hours were needed to finish the complete questionnaire. The intended learning objectives and the 4-point Likert scale in our study were based on the cognitive and psychomotor domain of Bloom’s taxonomy classification [12-14]. The participants were asked to evaluate the competencies by using the following 4-point Likert scale:

- 1 = Not Necessary
- 2 = Beginner - Theoretical knowledge: knowing terms/Practical Skills: knowledge of theory by practice
- 3 = Advanced - Theoretical knowledge: Being able to interpret/Practical Skills: perform under instruction by practice
- 4 = Expert - Theoretical knowledge: Being able to discuss intellectually/Practical Skills: perform independently

Additionally, an option “No Idea” was available, and responses of this option were excluded from further statistical analysis. The same Likert scale was used for all questions of the competencies, which made the scale a defensible approximation to an interval scale.

All data generated by this study were analyzed anonymously satisfying the EU Data Protection Directive 95/46/EC. The data protection officer of the first author’s university approved the study. The study was performed under the ethical regulations of that university.

### PHASE 3: Statistical evaluation

After 3 months the online survey was closed for contributions. The non-parametric Fisher’s Exact Test was applied after consulting by the statistical support service of the Institute of Biometrics of the University of Veterinary Medicine Hannover and the analysis was conducted with the statistic software SAS® (Version 9.2) under the assumption of unequal variances, two-tailed distributions and a significance level of 0.05. The results of free text questions were summarized and the three most mentioned objectives would be presented.

*Members of ECVN curriculum working group included H.A. Volk, J. Penderis, T.J. Anderson, S. Añor, A. Lujan-Feliu-Pascual, V.M. Stein and A. Tipold.

## Results

A return rate of 62% (n = 213/341) and the 77% overall complete response rate was achieved comprising 83 ESVN (including 46 residents) and 81 ECVN Diplomates. The respondents worked primarily in the United Kingdom (27%, n = 44), Germany (18%, n = 30), Italy (14%, n = 23) and Spain (9%, n = 15); 45% worked in academia, 44% in private specialty practice, 8% in both areas and 3% in industry or other organizations. 97% of respondents described themselves as primarily involved in small animal practice.

**Figure 1:**
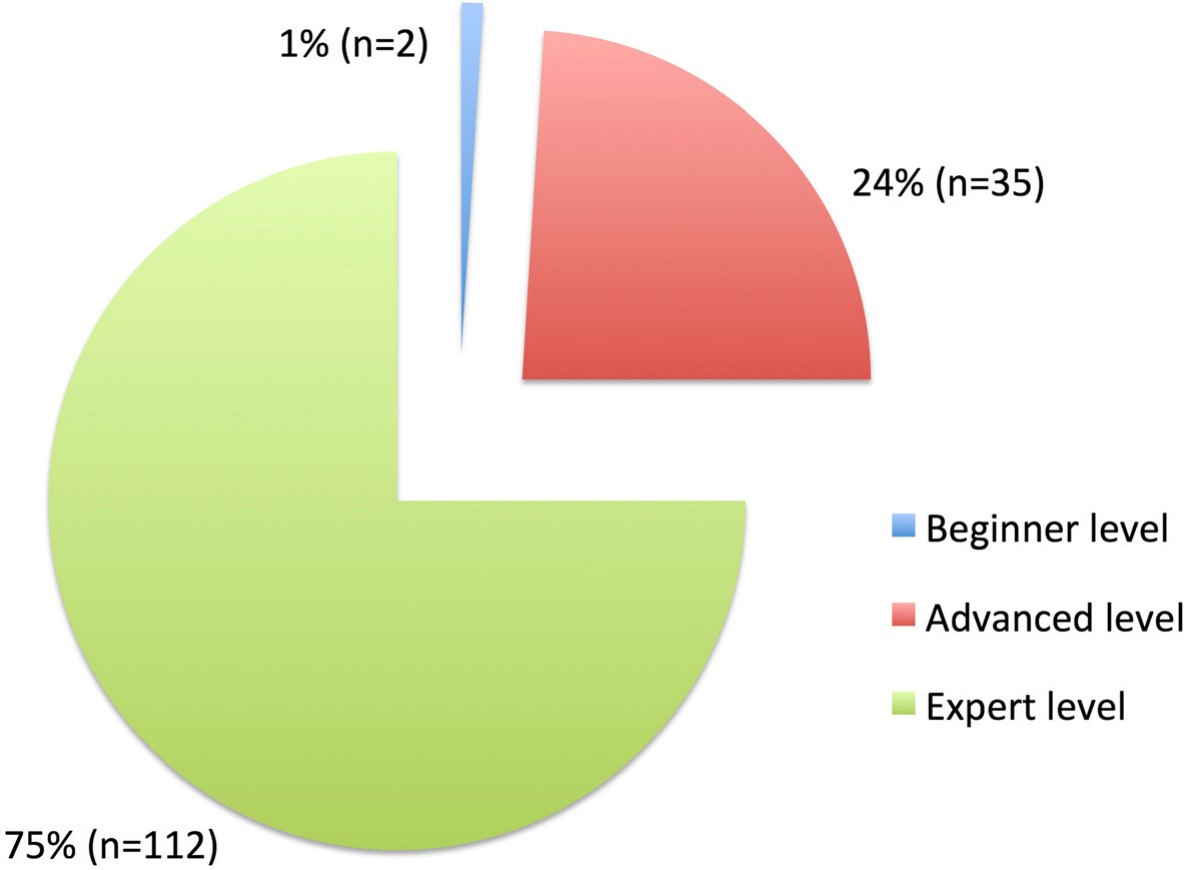
**Rating of the learning objectives all respondents.** Generally 75% (n = 112) of the competencies are expected to reach expert level, 24% (n = 35) in advanced level and only 1% (n = 2) in beginner level.

None of the suggested competencies were considered as “not necessary”, 75% (n = 112/149) of competencies were expected to attain expert level, 24% (n = 35/149) advanced level and 1% (n = 2/149) beginner level (Figure 1) (Additional file 1).

The 11 most important job competencies graded at expert level were confirmed (Table 1). Because of an equivalent rating for the last two competencies (mean rating 3.97), 11, instead of 10, competencies were listed comprising approximately the top 5% of all competencies. Items in the categories “Clinical examination” and “Disease Mechanisms” were overrepresented.

**Table 1:** **Rating of 11 most important job competencies**

**Competencies**	**Mean rating**
Anatomy and Physiology	
- Describe the gross neuroanatomic structures of the cat and dog brain and spinal cord	3.98
Clinical Methodology	
- Neurolocalize a lesion based on the examination findings	3.99
- Understand the risk factors and contraindications of CSF collection and methods to ameliorate these risks	3.99
- Perform cistern magna collection of CSF in the dog and cat	3.98
- Perform lumbar collection of CSF in the dog and cat	3.97
Disease mechanisms	
- Understand CNS diseases according to the VITAMIN-D principal	3.98
- Understand PNS diseases according to the VITAMIN-D principal	3.99
- Understand the pathogenesis of seizure disorders in dogs and cats.	3.97
- Understand the diagnosis and treatment of seizure disorders in dogs and cats	4
- Understand the pathogenesis of disc disease in dogs and cats.	3.99
- Understand the diagnosis and treatment of disc diseases in dog and cats	4

An advanced level of attainment was expected for competencies listed in the categories “Genetics and molecular biology” (100% (n = 7/7)), “Electrodiagnostic tests” (46% (n = 11/24)) and “Neuroradiology” (71% (n = 5/7)) (Additional file 1).

Experts highlighted 2 (1%) competencies for which they expected attainment at beginner level: “perform urinary tract electrodiagnostic testing in the dog and cat” and “apply radiation therapy technique” (Additional file 1).

Information summarizing the experience of respondents reflecting their experience of disease frequency and the most commonly used drugs was extracted from the free text questions. The three most important/most frequently seen diseases of the central nervous system (CNS) included epilepsy, intervertebral disc disease (IVDD), granulomatous meningoencephalitis (GME) (following hydrocephalus, fibrocartilaginous embolus (FCE) and steroid-responsive meningitis-arteritis (SRMA)); the three most important diseases of the peripheral nervous system (PNS) included polyradiculoneuritis, polyneuropathy, myasthenia gravis (following myositis, botulism and brachial plexus avulsion).

The three most frequently used immunosuppressive or anti-inflammatory drugs in veterinary neurology were glucocorticosteroids, azathioprine and cyclosporine; the three most frequently used antiepileptic drugs were benzodiazepine, phenobarbital, potassium bromide (following levetiracetam, gabapentin and zonisamide); the three most frequently used chemotherapeutic drugs groups included nitrosoureas, cytosine arabinoside and nitrogen mustards.

Comparisons of opinions were investigated between selected groups of respondents to test if there were biases in the answers:

1. ECVN Diplomates versus ESVN (Residents included) members.
2. Diplomates in academia versus Diplomates in private specialty practice.
3. Experts offering neurosurgery versus not offering neurosurgery.
4. Experience in neurology of 0–5, 6–10 and >10 years.

### Comparison between ECVN Diplomates and ESVN respondents

**Figure 2:**
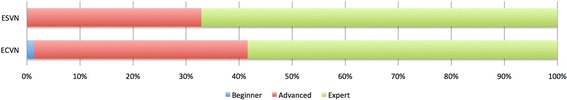
**Distribution of the expecting level from the groups ECVN and ESVN.** By ESVN, none of learning objectives would be considered as in beginner’s level, 33% (n = 49) as advanced and 67% (n = 100) as expert. By ECVN, 1% (n = 2) as beginner, 40% (n = 60) as advanced and 59% (n = 87) as expert.

Though 59% (n = 87/149) of the competencies reached expert level in both groups, a slight difference was found between the ECVN and ESVN group; ESVN members ranked 8% (n = 13) more competencies to reach expert level than ECVN Diplomates (Figure 2). In 22 competencies a significant different (p < 0,05) expectation was found between ECVN and ESVN. 12 (55%) of these competencies were in the area of electrodiagnostic tests and 8 (36%) in neuroradiology (Table 2).

**Table 2:** **Rating the importance of competencies by comparing the groups ESVN and ECVN members; these members rated 22 learning objectives significantly different**

	**Mean ECVN**	**Mean ESVN**	**P-value**
**Genetics and molecular biology**			
Understand the principles of errors of cellular metabolism	3,01	**3,26**	0,0143
**Clinical methodology**			
**> EEG**			
Perform EEG testing in the dog and cat	2,91	**3,42**	P < 0.0001
Interpret EEG testing in the dog and cat	2,96	**3,45**	P < 0.0001
**> EMG**			
Perform EMG and nerve conduction testing in the horse.	3	**3,35**	0,0224
Perform single fiber EMG testing in the dog and cat.	2,58	**3,15**	0,001
Interpret single fiber EMG testing in the dog and cat.	2,93	**3,35**	0,0183
**> SSEP**			
Perform somatosensory evoked potential testing in the dog and cat	2,85	**3,39**	P < 0.0001
Interpret somatosensory evoked potential testing in the dog and cat	3,08	**3,55**	P < 0.0001
**> OEA**			
Perform otoacoustic emission testing in the dog and cat	2,53	**3,07**	0,0017
Interpret otoacoustic emission testing in the dog and cat	2,73	**3,25**	0,0043
**> VEP**			
Perform visual evoked potential testing in the dog and cat	2,48	**2,95**	0,009
Interpret visual evoked potential testing in the dog and cat	2,67	**3,12**	0,0092
**> Urinary tract electro. Testing**			
Perform urinary tract electrodiagnostic testing in the dog and cat	2,25	**2,63**	0,0282
**Neuroradiology**			
**> Theory**			
Understand CT scanning technique	3,65	**3,67**	0,0386
Understand CT physics	3,1	**3,15**	0,0488
Understand MRI scanning technique	**3,64**	3,6	0,0011
Understand MRI physics	3,03	**3,25**	0,0047
Understand nervous system ultrasound technique	2.96	**3.23**	0.0368
**> Practical**			
Perform myelography in the horse	2,63	**3,12**	0,0036
Apply radiation therapy technique	2,16	**2,56**	0,0339
**Academia competencies**			
In laboratorium	2,94	**3,27**	0,023
In epidemiology	2,87	**3,24**	0,0204

Values in boldface have higher mean rating.

### Comparison between diplomates in academia and in specialty practice

**Figure 3:**
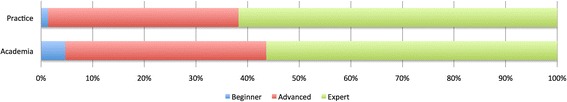
**Rating the importance of competencies by comparing the groups diplomates in academia and in specialty practice; these members rated 3 learning objectives significantly different.** By diplomates in practice, only 1% of learning objectives would be considered as in beginner’s level, 37% (n = 55) as advanced and 62% (n = 92) as expert. By diplomates in acadmia, 5% (n = 7) as beginner, 39% (n = 58) as advanced and 56% (n = 84) as expert.

In 56% (n = 84/149) of the competencies both groups agreed on reaching expert level (Figure 3). A slight difference (p < 0,05) in the opinion of these two groups was detected. 6% more job competencies were expected from diplomates in specialty practice to reach expert level. In contrast, seven competencies were considered only to require the attainment of a beginner’s level of competencies by the group working in academia. In four competencies significant differences between the two groups were found (Table 3).

**Table 3:** **Rating the importance of competencies by comparing the groups diplomates in academia and in specialty practice; these members rated 3 learning objectives significantly different**

**Competencies**	**Diplomates in academia**	**Diplomates in practice**	**P-value**
**Clinical methodology**			
Interpret urinary tract electrodiagnostic testing in the dog and cat	2,4	**2,71**	0,0085
**Neuroanaesthesia & Neurosurgery**			
Perform atlantoaxial subluxation fixation techniques	3,17	**3,56**	0,0236
**Pathology**			
Understand basic PNS pathological interpretation	3,48	**3,77**	0,0394
Understand microscopic pathological features of specific small animal diseases	3,47	**3,6**	0,0114

Values in filled cells have higher mean rating.

### Comparison between experts offering neurosurgery and not-offering neurosurgery

**Figure 4:**
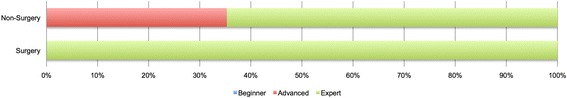
**Distribution of expecting levels from the groups “offering surgery” and “non-offering surgery”.** By group “Non-offering surgery”, none of learning objectives would be considered as in beginner’s level, 35% (n = 6) as advanced and 65% (n = 11) as expert. By group “offering surgery”, 100% (n = 17) as expert.

The comparisons between these two groups were limited to the 17 job competencies included in the categories “Neuroanaesthesia and Neurosurgery”. Experts offering neurosurgery expected residents to attain an expert level in all job competencies in the category “Neuroanaesthesia and Neurosurgery”. In contrast, experts not offering neurosurgery considered only 65% (n = 11/17) of these competencies to were required to attain expert level (Figure 4). Ten competencies showed significant differences between both groups, 9 of these received higher mean ratings from experts who were performing neurosurgery (Table 4).

**Table 4:** **In 10 competencies significant differences were detected between the groups “offering surgery” and “not-offering surgery”**

	**Mean non-surgery**	**Mean surgery**	**P-value**
**Neuroanaesthesia & Neurosurgery**			
**> Theory**			
Understand fluid therapy for a neurological patient	**3,91**	3,73	0.0241
**> Practical**			
Perform ventral slot	3.67	**3.89**	0.0014
Perform thoracolumbar hemilaminectomy	3.7	**3.93**	P < 0.0001
Perform dorsal laminectomy of cervical spine	3.59	**3.79**	0.0212
Perform fenestration	3.65	**3.9**	P < 0.0001
Perform ventriculo-peritoneal shunt	3.22	**3.51**	0.0306
Perform craniotomy/craniectomy	3.3	**3.62**	0.01
Perform fracture repair	3.22	**3.53**	0.0133
Perform dorsal laminectomy of lumbosacral spine	3.52	**3.8**	0.0018
Perform atlantoaxial subluxation fixation techniques	3.26	**3.57**	0.0331

Values in boldface have higher mean rating.

### Comparison of respondents responses according to the experience in the practice of neurology of 0–5, 6–10 and >10 years

**Figure 5:**
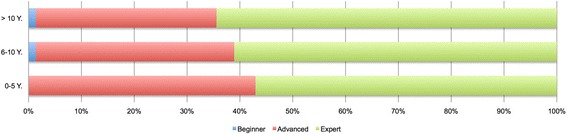
**Distribution of expecting levels from the groups with different experience in neurology (0–5, 6–10 and >10 years).** By experience with 0–5 years, none of learning objectives would be considered as beginner, 43% (n = 64) as advanced and 57% (n = 85) as expert. By 6–10 years, 1% (n = 2) as beginner, 38% (n = 56) as advanced and 61% (n = 91) as expert. By >10 years, 1% (n = 2) as beginner, 34% (n = 51) as advanced and 65% (n = 96) as advanced.

For all three groups a similar pattern in the distribution of the expected levels of attainment was observed. However, the respondents with greater than ten years of experience in neurology expected more competencies to reach expert level than the other two groups (Figure 5). Significant differences were found in 11 competencies between groups with 0–5 and 6–10 years experience, 9 competencies between groups with 6–10 and >10 years experience, and 16 between groups with 0–5 and >10 years experience (Table 5).

**Table 5:** **Competencies with significant difference between the groups with different experience in neurology (0–5, 6–10, >10 years)**

	**Mean 0-5**	**Mean 6-10**	**Mean >10**	**P-values**
**Pharmacology and toxicology**				
**> pharmacodynamic and pharmacokinetic**				
Understand the major neurotransmitters and their receptors of the central and peripheral nervous system	3.79*	3.74*	3.76	0.0269*
Understand the difference between pharmacokinetics and pharmacodynamic qualities of drugs and the parameters used to quantify these qualities	3.28***	3.26	3.52***	0.0361***
**> Chemotherapeutic drugs**				
Understand the pharmacokinetics of chemotherapeutic drugs for nervous system neoplasia/Inflammation	3.35***	3.4	3.52***	0.0328***
**Genetics and molecular biology**				
Understand the difference between transcription versus translation	3	2.81**	3.13**	0.0439**
Understand the genome organization and chromosome structure	2.85	2.81**	3.11**	0.0405**
Understand the inheritance patterns and types of mutations	3.13	3.07**	3.42**	0.022**
Understand the principles of common molecular genetic tools (laboratory methods; SNPs, microsatellite mapping, candidate genes)	2.78	2.65**	3.08**	0.0146**
Understand how to investigate a breed related disorder for an underlying genetic mutation	3.21*	3.33*	3.42	0.0384*
**Clinical methodology**				
**> CSF**				
Interpret laboratory results of CSF	3.91***	3.95	4***	0.0424***
**> EEG**				
Perform EEG testing in the dog and cat	3.38* ***	3.05*	3.07***	0.0128* 0.0459***
Interpret EEG testing in the dog and cat	3.4* ***	3.09*	3.12***	0.0063* 0.0187***
**> EMG**				
Perform single fiber EMG testing in the dog and cat.	3.14*	2.6*	2.82	0.0041*
**> Urinary tract electro. Testing**				
Interpret urinary tract electrodiagnostic testing in the dog and cat	2.81***	2.43	2.79***	0.0345***
**> Bone marrow aspiration & core biopsy**				
Interpret results of bone marrow aspirate and or core biopsy	2.82	2.74**	2.97**	0.0242**
**Disease mechanisms**				
**> Disc diseases**				
Understand the pathogenesis of disc diseases in horses	3.43***	3.35	3.29***	0.0342***
**> Micturition disorders**				
Understand the pathogenesis of Micturition disorders in ruminants/food animals	3.21***	3	2.97***	0.0377***
**Neuroanaesthesia & Neurosurgery**				
Understand anesthesia of the neurological patient	3.64	3.52**	3.74**	0.0023**
Understand peri-operative antibiotic recommendations	3.68***	3.71	3.87***	0.0461***
Perform ventral slot	3.9***	3.83	3.73***	0.036***
**Neuroradiology**				
Understand CT scanning technique	3.55***	3.62**	3.77** ***	0.0154** 0.0019***
Understand nervous system ultrasound technique	2.96*	3.1*	3.2	0.0484*
Understand nuclear medicine technique	2.73***	2.74	2.95***	0.0386***
Interpret radiographs of the skull	3.7***	3.78	3.89***	0.0032***
**Pathology**				
Exhibit competence in CSF cytological interpretation in small animals	3.64* ***	3.88*	3.89***	0.0188* 0.0345***
Exhibit competence in CSF sample examination (protein content, cell counting)	3.53* ***	3.88*	3.68***	0.0273* 0.0134***
Understand microscopic pathological features of specific horse diseases	3.15*	3.18*	3.17	0.0262*
Understand Infectious disease testing techniques (PCR/Western blot/Serology)	3.11***	3.05**	3.4** ***	0.0248** 0.0052***
**Academia Competencies**				
In teaching for undergraduates	3.55*	3.88*	3.63	0.0126*
In teaching for postgraduates	3.53*	3.85*	3.69	0.0092*
In statistics	3.02	2.87**	3.11**	0.0306**

Values with *, indicate the significance of the learning objectives between groups 0-5 and 6-10; for groups 6-10 and >10 are indicated with **; and groups 0-5 and >10 with ***.

### Difference between diplomates and advanced practitioners

All responders expected that diplomates have a detailed understanding of veterinary neurology in a clinical setting, an advanced level in research as well as competencies in teaching, while advanced practitioners were not expected to have competencies in teaching and research.

## Discussion

Although the whole questionnaire included 149 4-point Likert scale questions and 49 questions in free text form and about 90 minutes were needed to complete it, the response rate was still satisfactory with a 62% return rate and a 48% overall response rate. Experts in veterinary neurology seem to have a genuine interest in teaching matters.

In contrast to Part I (LIN, Y.-W., VOLK, H., PENDERIS, J., TIPOLD, A., & EHLERS, J. P.: Development of learning objectives for neurology in a veterinary curriculum: Part I: undergraduates: BMC Vet Res 2015, 11:2.), where undergraduates were expected to reach beginner level in 71% of learning objectives and 26% of learning objectives were considered as “not necessary”, diploma holders of the ECVN were expected to reach experts level in 75% of competencies and none of them was regarded as “not necessary”. Neurology education for undergraduates requires a minimum body of clinical neurology knowledge and skills, without considering their eventual career path [15]. For postgraduate training of specialists, requirements of knowledge and skills are expected to be on a much higher level as also shown in the current study. Answers from residents were included in the “ESVN group” in this study, because they have a heterogeneous level of knowledge (1–3 years residents) and the trainer status of both ESVN members and residents might be similar.

Surprisingly, in the current study a difference in opinion was seen between diplomates working in academia and diplomates working in private specialty practice. Experts working in private specialty practice expected for all competencies, which differed significantly, higher mean rating than experts in academia. Similar observations occurred also by comparison between ESVN and ECVN members. Experts of the ESVN expected in 21 of 22 significant learning objectives also higher mean rating than ECVN diplomates.

This phenomenon might be explained by the different focus of these groups. Diplomates in academia have a multitude of tasks and have to find a balance between working on clinical cases, undergraduate and postgraduate student education, administration and research. In most clinics of universities several specialists treat single animals trans-disciplinarily. In contrast, diplomates in private specialty practice focus more on expanding their competencies to manage a bigger variety of patients independently. This difference in the job description may explain the slightly higher expectation in clinical knowledge and skills.

Surprisingly, the learning objectives in the field of “electrodiagnostic tests” received controversial results. In total only 35 competencies were expected to reach an advanced level and 11 (31%) of these competencies were in the electrodiagnostic tests category (Additional file 1). Moreover, 12 of 22 (55%) learning objectives, which showed significant differences between ESVN and ECVN experts, were also in the electrodiagnostic tests category. According to this result the college has to discuss the depth of education and outcome evaluation in this specific field of neurology.

Besides the requirement of reaching the highest standard in the field of clinical neurology and research, ECVN diplomates are expected to possess teaching competencies. These competencies may distinguish the job specification of diplomates and advanced practitioners. Veterinary Continuous Education in Europe (VetCEE) assigned a role for diplomates as trainers in postgraduate education of veterinarians in whole Europe [16]. The competencies of teaching should therefore also be part of a residency training for future diplomates, as the neurologist William A. Pulsinelli expressed: “Residents are encouraged to teach the teacher and thereby enrich everyone’s education” [17].

In the current study the six most important disease processes of the CNS and PNS were defined. Also the three most frequently used immunosuppressive, antiepileptic and chemotherapeutic drugs were extracted from free text questions. Knowledge about such diseases and drugs may be considered as part of the examination content [18].

Using the Delphi-method made it possible to collect objectively the opinion of learners (residents; “learner-centered”) and trainers (diplomates; “teacher-centered”), which can be used for curriculum adaption. The learner-centered focus includes frequently the need, skills and interests of the learner, which is often accompanied by a problem-based approach providing active learning and high motivation [19]. The opinions of residents from the questionnaire should be therefore considered in the design of resident training.

A limitation of the current study is the limited consideration of affective elements, one of the three domains of educational objectives in Bloom’s taxonomy [12]. In the field of medicine, communication skills, dealing with ethical matters and inter-professional relations are important [20]. Such professional attitudes have received increasing attention in recent years, while traditionally only little attention in medical education was perceived [21]. For a better and safe practice, “The Good Medical Practice” (GMP) from the General Medical Council of United Kingdom has set certain standards expected of all doctors [22,23]. Also the glossary of terms from Accreditation Council for Graduate Medical Education (ACGME) defines that “competencies” are not only confined to specific knowledge and skills, but should also include behavior and attitudes in graduate medical education [24]. In the future, the affective elements in veterinary postgraduate education should be also investigated and assessed, but could be part of a general training provided by all specialty colleges.

## Conclusion

The results of our study confirmed the hypothesis that the majority of competencies are expected to reach expert level for ECVN diplomates to meet their job requirements. According to these job requirements of ECVN diplomates, residents as future diplomats are expected in addition to advancing scientifically knowledge and clinical skills 1) to have completed a well-structured training program of adequate length under direct supervision, 2) to be active in education and 3) to be able to interact and communicate in a professional manner with a variety of stakeholders, including other experts and the public. The taxonomic catalog of learning objectives in the current study could be used by the ECVN to adapt their postgraduate curriculum. Moreover, regarding the continually developing of veterinary specialism, a periodic reevaluation of competencies should be conducted to guarantee the up to date status of the curriculum.

## Additional files

Additional file 1:
**Learning objectives with mean values and level distribution for Residents/Diplomates of the European College of Veterinary Neurology.**
Additional file 2:
**Difference between ECVN Diplomates and advanced practitioners.**

## Abbreviations

ACGME: Accreditation Council for Graduate Medical Education
ACVIM: American College of Veterinary Internal Medicine
CNS: Central nervous system
EBVS: Board of Veterinary Specialisation
ECVN: European College of Veterinary Neurology
ESVN: European Society of Veterinary Neurology
FCE: Fibrocartilaginous Embolus
GME: Granulomatous Meningoencephalitis
GMP: Good medical practice
IVDD: Intervertebral disc disease
PNS: Peripheral nervous system
SRMA: Steroid-responsive meningitis-arteritis
VetCEE: Veterinary Continuous Education in Europe
WSAVA: World Small Animal Veterinary Association
