# Supplementary material for: Development of learning objectives for neurology in a veterinary curriculum: Part II: Postgraduates
Source: BMC Vet Res. 2015 Jan 27;11:10. doi: 10.1186/s12917-014-0314-4 (PMC4323235; doi:10.1186/s12917-014-0314-4)
Supplement: Additional file 1: — Learning objectives with mean values and level distribution for Residents/Diplomates of the European College of Veterinary Neurology. [file 12917_2014_314_MOESM1_ESM.doc]

**Development of learning objectives for neurology in a veterinary curriculum. Part II: Postgraduates**

**Additional file 1**Learning objectives with mean values and level distribution for Residents/Diplomates of the European College of Veterinary Neurology.

| **Learning Objectives1** | **N** | **B** | **A** | **E** |
| --- | --- | --- | --- | --- |
| N = Not Necessary (blue) (1 - 1.44) |  |  |  |  |
| B = Beginner Level (green) (1.45 - 2.44) |
| A = Advanced Level (orange) (2.45-3.44) |
| E = Expert Level (red) (3.45 - 4) |
| **Anatomy and Physiology** |  |  |  |  |
| 1. Understand the gross neuroanatomic structures of the cat and dog brain and spinal cord |  |  |  | 3.98 |
| 2. Understand the microscopic anatomy of the nervous system |  |  |  | 3.72 |
| 3. Understand the functional neuroanatomy of the central nervous system |  |  |  | 3.96 |
| 4. Understand the functional neuroanatomy of the peripheral nervous system |  |  |  | 3.96 |
| 5. Understand the functional neuroanatomy of the autonomic nervous system |  |  |  | 3.86 |
| 6. Understand the basic principles of neurophysiology in regards to membrane potentials, action potential generation, ion channel conductance and synaptic neurotransmission in the central and peripheral nervous system |  |  |  | 3.79 |
| 7. Understand the principles of cerebrospinal fluid dynamics and intracranial pressure |  |  |  | 3.94 |
| * What other aspects of Anatomy and Physiology (besides those listed above) do you think should form part of the ECVN Residency Training? |  |  |  |  |
|  |  |  |  |  |
| **Pharmacology and Toxicology** |  |  |  |  |
| **> pharmacodynamic and Pharmacokinetic** |  |  |  |  |
| 1. Understand the autonomic nervous system receptors and neurotransmitters |  |  |  | 3.74 |
| 2. Understand the major neurotransmitters and their receptors of the central and peripheral nervous system |  |  |  | 3.76 |
| 3. Understand the mechanisms of drug- delivery through the blood-brain barrier |  |  |  | 3.72 |
| 4. Understand the basic principles of drug absorption, metabolism and clearance |  |  |  | 3.65 |
| 5. Understand the difference between pharmacokinetics and pharmacodynamic qualities of drugs and the parameters used to quantify these qualities |  |  | 3.37 |  |
| 6. Understand therapeutic index in relation to drug efficacy and safety |  |  |  | 3.52 |
| * What other aspects of pharmacodynamic & pharmacokinetic (besides those listed above) do you think should form part of the ECVN Residency Training? |  |  |  |  |
| **> Pain** |  |  |  |  |
| 7. Understand the principles of pain activation pathways |  |  |  | 3.79 |
| 8. Understand the mechanism of action of pain therapy |  |  |  | 3.8 |
| * What other aspects of pain (besides those listed above) do you think should form part of the ECVN Residency Training? |  |  |  |  |
| **> Neurotoxin** |  |  |  |  |
| 9. Understand the major classes of neurotoxins and the effect on the nervous system |  |  |  | 3.79 |
| * What other aspects of neurotoxin (besides those listed above) do you think should form part of the ECVN Residency Training? |  |  |  |  |
| **> Antiepileptic drugs** |  |  |  |  |
| 10. Understand the mechanism of the major classes of anti-epileptic drugs |  |  |  | 3.96 |
| 11. Understand the pharmacokinetics of anti- epileptic drugs |  |  |  | 3.86 |
| 12. Understand the side-effect profiles of anti-epileptic drugs |  |  |  | 3.94 |
| * What do you think are the 10 most important antiepileptic drugs currently in use in veterinary neurology practice? |  |  |  |  |
| **> Immunosuppression** |  |  |  |  |
| 13. Understand the mechanism of immunosuppressive drugs for CNS inflammatory disease |  |  |  | 3.75 |
| 14. Understand the pharmacokinetics of immunosuppressive drugs for CNS inflammatory disease |  |  |  | 3.56 |
| 15. Understand the side-effect profiles of the immunosuppressive drugs for CNS inflammatory disease |  |  |  | 3.81 |
| * What do you think are the 10 most important immunosuppressive drugs currently in use in veterinary neurology practice? |  |  |  |  |
| **> Chemotherapeutic drugs** |  |  |  |  |
| 16. Understand the mechanism of chemotherapeutic drugs for nervous system neoplasia / inflammation |  |  |  | 3.55 |
| 17. Understand the pharmacokinetics of chemotherapeutic drugs for nervous system neoplasia / Inflammation |  |  | 3.43 |  |
| 18. Understand the side-effects profiles of chemotherapeutic drugs for nervous system neoplasia / inflammation |  |  |  | 3.67 |
| * What do you think are the 10 most important chemotherapeutic drugs currently in use in veterinary neurology practice? |  |  |  |  |
|  |  |  |  |  |
| **Genetics and Molecular Biology** |  |  |  |  |
| 1. Understand the structure of DNA and a gene |  |  | 3.17 |  |
| 2. Understand the difference between transcription versus translation |  |  | 3.01 |  |
| 3. Understand the genome organization and chromosome structure |  |  | 2.94 |  |
| 4. Understand the inheritance patterns and types of mutations |  |  | 3.23 |  |
| 5. Understand the principles of common molecular genetic tools (laboratory methods; SNPs, microsatellite mapping, candidate genes) |  |  | 2.86 |  |
| 6. Understand the principles of errors of cellular metabolism |  |  | 3.13 |  |
| 7. Understand how to investigate a breed related disorder for an underlying genetic mutation |  |  | 3.32 |  |
| * What other aspects of genetics and molecular biology (besides those listed above) do you think should form part of the ECVN Residency Training? |  |  |  |  |
|  |  |  |  |  |
| **Clinical Methodology** |  |  |  |  |
| **> Neurologic Examination** |  |  |  |  |
| 1. Perform a neurologic examination of all species |  |  |  | 3.94 |
| 2. Neurolocalize a lesion based on the examination findings |  |  |  | 3.99 |
| * What other aspects of neurologic examination (besides those listed above) do you think should form part of the ECVN Residency Training? |  |  |  |  |
| **> Laboratory** |  |  |  |  |
| 3. Interpret hematological, serum chemistry and urinalysis results |  |  |  | 3.88 |
| 4. Understand organ function tests (liver, endocrine) |  |  |  | 3.85 |
| 5. Interpret organ function tests (liver, endocrine) |  |  |  | 3.83 |
| * What other aspects of laboratory (besides those listed above) do you think should form part of the ECVN Residency Training? |  |  |  |  |
| **> CSF** |  |  |  |  |
| 6. Understand the risk factors and contraindications of CSF collection and methods to ameliorate these risks. |  |  |  | 3.99 |
| 7. Perform cistern magna collection of CSF in the dog and cat |  |  |  | 3.98 |
| 8. Perform lumbar collection of CSF in the dog and cat |  |  |  | 3.97 |
| 9. Perform lumbar CSF collection in the horse / ruminant / food animal |  |  | 3.39 |  |
| 10. Interpret laboratory results of CSF |  |  |  | 3.96 |
| * What other aspects of cerebrospinal fluid (besides those listed above) do you think should form part of the ECVN Residency Training? |  |  |  |  |
| **Electrodiagnostic tests** |  |  |  |  |
| * Which electrodiagnostic tests, procedures or investigations do you perform in your neurology practice? |  |  |  |  |
| **> EEG** |  |  |  |  |
| 11. Perform EEG testing in the dog and cat |  |  | 3.16 |  |
| 12. Interpret EEG testing in the dog and cat |  |  | 3.2 |  |
| * What other aspects of EEG (besides those listed above) do you think should form part of the ECVN Residency Training? |  |  |  |  |
| **> EMG** |  |  |  |  |
| 13. Perform EMG and nerve conduction testing in the dog and cat |  |  |  | 3.93 |
| 14. Interpret EMG and nerve conduction testing in the dog and cat. |  |  |  | 3.93 |
| 15. Perform F-waves, Repetitive stimulation and H-wave testing in the dog and cat. |  |  |  | 3.71 |
| 16. Interpret F-waves, Repetitive stimulation and H-wave testing in the dog and cat. |  |  |  | 3.81 |
| 17. Perform EMG and nerve conduction testing in the horse. |  |  | 3.17 |  |
| 18. Interpret EMG and nerve conduction testing in the horse. |  |  | 3.36 |  |
| 19. Perform EMG and nerve conduction testing in the ruminant / food animal . |  |  | 2.92 |  |
| 20. Interpret EMG and nerve conduction testing in the ruminant / food animal |  |  | 3.16 |  |
| 21. Perform single fiber EMG testing in the dog and cat. |  |  | 2.86 |  |
| 22. Interpret single fiber EMG testing in the dog and cat. |  |  | 3.13 |  |
| * What other aspects of EMG (besides those listed above) do you think should form part of the ECVN Residency Training? |  |  |  |  |
| **> SSEP (SOMATOSENSORY EVOKED POTENTIAL TESTING)** |  |  |  |  |
| 23. Perform somatosensory evoked potential testing in the dog and cat |  |  | 3.11 |  |
| 24. Interpret somatosensory evoked potential testing in the dog and cat |  |  | 3.3 |  |
| * What other aspects of somatosensory evoked potential testing (besides those listed above) do you think should form part of the ECVN Residency Training? |  |  |  |  |
| **> BAER (AUDIOTORY EVOKED POTENTIAL TESTING)** |  |  |  |  |
| 25. Perform brainstem auditory evoked potential testing in the dog and cat |  |  |  | 3.84 |
| 26. Interpret brainstem auditory evoked potential testing in the dog and cat |  |  |  | 3.9 |
| * What other aspects of audiotory evoked potential testing (besides those listed above) do you think should form part of the ECVN Residency Training? |  |  |  |  |
| **> OEA (OTOACOUSTIC EMISSION TESTING)** |  |  |  |  |
| 27. Perform otoacoustic emission testing in the dog and cat |  |  | 2.78 |  |
| 28. Interpret otoacoustic emission testing in the dog and cat |  |  | 2.98 |  |
| * What other aspects of otoacoustic emission testing (besides those listed above) do you think should form part of the ECVN Residency Training? |  |  |  |  |
| **> VEP (VISUAL EVOKED POTENTIAL TESTING)** |  |  |  |  |
| 29. Perform visual evoked potential testing in the dog and cat |  |  | 2.7 |  |
| 30. Interpret visual evoked potential testing in the dog and cat |  |  | 2.89 |  |
| * What other aspects of visual evoked potential testing (besides those listed above) do you think should form part of the ECVN Residency Training? |  |  |  |  |
| **> URINARY TRACT ELECTRO. TESTING** |  |  |  |  |
| 31. Perform urinary tract electrodiagnostic testing in the dog and cat |  | 2.43 |  |  |
| 32. Interpret urinary tract electrodiagnostic testing in the dog and cat |  |  | 2.69 |  |
| * What other aspects of urinary tract electro. testing (besides those listed above) do you think should form part of the ECVN Residency Training? |  |  |  |  |
| **> OPHTAMOLOGIC ELECTRO. TESTING** |  |  |  |  |
| 33. Perform ophthalmologic electrodiagnostic testing (ERG, VEP) in the dog and cat. |  |  | 2.68 |  |
| 34. Interpret ophthalmologic electrodiagnostic testing (ERG, VEP) in the dog and cat |  |  | 2.89 |  |
| * What other aspects of ophtalmologic electro. testing (besides those listed above) do you think should form part of the ECVN Residency Training? |  |  |  |  |
| **> BONE MARROW ASPIRATION & CORE BIOPSY** |  |  |  |  |
| 35. Perform a bone marrow aspirate and or core biopsy |  |  | 2.83 |  |
| 36. Interpret results of bone marrow aspirate and or core biopsy |  |  | 2.85 |  |
| * What other aspects of bone marrow aspiration and core biopsy (besides those listed above) do you think should form part of the ECVN Residency Training? |  |  |  |  |
|  |  |  |  |  |
| **Disease Mechanisms** |  |  |  |  |
| **> CNS** |  |  |  |  |
| 1. Understand CNS diseases according to the VITAMIN-D principal |  |  |  | 3.98 |
| *2. Please list the 10 most important diseases of CNS that a Residents or an ECVN Diplomates should know. |  |  |  |  |
| *3. What other aspects of diseases of CNS (besides those listed above) do you think should form part of the ECVN Residency Training? |  |  |  |  |
| **> PNS** |  |  |  |  |
| 4. Understand PNS diseases according to the VITAMIN-D principal |  |  |  | 3.99 |
| *5. Please list the 10 most important diseases of PNS that a Residents or an ECVN Diplomates should know. |  |  |  |  |
| *6. What other aspects of diseases of PNS (besides those listed above) do you think should form part of the ECVN Residency Training? |  |  |  |  |
| **> Seizure** |  |  |  |  |
| 7. Understand the pathogenesis of seizure disorders in dogs and cats. |  |  |  | 3.97 |
| 7.1 Understand the the diagnosis and treatment of seizure disorders in dogs and cats |  |  |  | 4 |
| 8. Understand the pathogenesis of seizure disorders in horses. |  |  |  | 3.59 |
| 8.1 Understand the diagnosis and treatment of seizure disorders in horses |  |  |  | 3.65 |
| 9. Understand the pathogenesis of seizure disorders in ruminants / food animals. |  |  | 3.36 |  |
| 9.1 Understand the diagnosis and treatment of seizure disorers in ruminants / food animals |  |  | 3.35 |  |
| * What other aspects of seizure (besides those listed above) do you think should form part of the ECVN Residency Training? |  |  |  |  |
| **> Disc Disease** |  |  |  |  |
| 10. Understand the pathogenesis of disc diseases in dogs and cats. |  |  |  | 3.99 |
| 10.1 Understand the diagnosis and treatment of disc diseases in dogs and cats |  |  |  | 4 |
| 11. Understand the pathogenesis of disc diseases in horses. |  |  | 3.34 |  |
| 11.1 Understand the diagnosis and treatment of disc diseases in horses |  |  | 3.36 |  |
| * What other aspects of disc diseases (besides those listed above) do you think should form part of the ECVN Residency Training? |  |  |  |  |
| **> Micturition Disorders** |  |  |  |  |
| 12. Understand the pathogenesis of micturition disorders in dogs and cats. |  |  |  | 3.92 |
| 12.1 Understand the diagnosis and treatment of micturition disorders in dogs and cats |  |  |  | 3.94 |
| 13. Understand the pathogenesis of micturition disorders in horses. |  |  | 3.33 |  |
| 13.1 Understand the diagnosis and treatment of micturition disorders in horses |  |  | 3.28 |  |
| 14. Understand the pathogenesis of micturition disorders in ruminants / food animals |  |  | 3.05 |  |
| 14.1 Understand the diagnosis and treatment of micturition disorders in ruminants / food animals |  |  | 3.06 |  |
| * What other aspects of micturition disorders (besides those listed above) do you think should form part of the ECVN Residency Training? |  |  |  |  |
|  |  |  |  |  |
| **Neuroanaesthesia & Neurosurgery** |  |  |  |  |
| **> Theory** |  |  |  |  |
| 1. Understand anesthesia of the neurological patient |  |  |  | 3.65 |
| 2. Understand fluid therapy for a neurological patient |  |  |  | 3.78 |
| 3. Understand peri-operative antibiotic recommendations |  |  |  | 3.76 |
| 4. Understand tissue handling theory and techniques (Biopsies) |  |  |  | 3.77 |
| * What other aspects of neuroanaesthesia and neurosurgery - THEORY (besides those listed above) do you think should form part of the ECVN Residency Training? |  |  |  |  |
| **> Practical** |  |  |  |  |
| 5. Perform ventral slot |  |  |  | 3.82 |
| 6. Perform thoracolumbar hemilaminectomy |  |  |  | 3.85 |
| 7. Perform dorsal laminectomy of cervical spine |  |  |  | 3.72 |
| 8. Perform fenestration |  |  |  | 3.82 |
| 9. Perform cervical vertebral distraction- fusion |  |  |  | 3.47 |
| 10. Perform ventriculo-peritoneal shunt |  |  | 3.41 |  |
| 11. Perform craniotomy / craniectomy |  |  |  | 3.51 |
| 12. Perform brain biopsy |  |  | 3.41 |  |
| 13. Perform fracture repair |  |  | 3.42 |  |
| 14. Perform dorsal laminectomy of lumbosacral spine |  |  |  | 3.7 |
| 15. Perform atlantoaxial subluxation fixation techniques |  |  |  | 3.47 |
| 16. Perform muscle biopsy |  |  |  | 3.9 |
| 17. Perform nerve biopsy |  |  |  | 3.88 |
| * What other aspects of neuroanaesthesia and neurosurgery - PRACTICAL (besides those listed above) do you think should form part of the ECVN Residency Training? |  |  |  |  |
| * Which neurosurgical procedures do you perform in your neurology practice? |  |  |  |  |
|  |  |  |  |  |
| **Neuroradiology** |  |  |  |  |
| **> Theory** |  |  |  |  |
| 1. Understand CT scanning technique |  |  |  | 3.66 |
| 2. Understand CT physics |  |  | 3.13 |  |
| 3. Understand MRI scanning technique |  |  |  | 3.62 |
| 4. Understand MRI physics |  |  | 3.14 |  |
| 5. Understand nervous system ultrasound technique |  |  | 3.1 |  |
| 6. Understand nuclear medicine technique |  |  | 2.83 |  |
| 7. Understand radiation therapy principles |  |  | 3.11 |  |
| * What other aspects of neuroradiology - THEORY (besides those listed above) do you think should form part of the ECVN Residency Training? |  |  |  |  |
| **> Practical** |  |  |  |  |
| 8. Interpret radiographs of the abdomen and thorax |  |  |  | 3.53 |
| 9. Interpret radiographs of the axial and appendicular skeleton |  |  |  | 3.79 |
| 10. Interpret radiographs of the skull |  |  |  | 3.8 |
| 11. Interpret CT scan of the brain and skull |  |  |  | 3.93 |
| 12. Interpret CT scan of the vertebral column and spinal cord |  |  |  | 3.95 |
| 13. Interpret MRI scans of the brain |  |  |  | 3.96 |
| 14. Interpret MRI scans of the spine |  |  |  | 3.96 |
| 15. Interpret MRI scans of the peripheral nervous system |  |  |  | 3.8 |
| 16. Identify the different MRI scan techniques and their use in clinical practice |  |  |  | 3.78 |
| 17. Interpret myelograms in the cat, dog and horse |  |  |  | 3.87 |
| 18. Perform myelography in the dog and cat |  |  |  | 3.83 |
| 19. Perform myelography in the horse |  |  | 2.88 |  |
| 20. Perform nervous system ultrasound interpretation |  |  | 2.78 |  |
| 21. Perform nuclear medicine interpretation |  |  | 2.63 |  |
| 22. Apply radiation therapy technique |  | 2.36 |  |  |
| * What other aspects of neuroradiology - PRACTICAL (besides those listed above) do you think should form part of the ECVN Residency Training? |  |  |  |  |
| * Which neuroradiology procedures do you perform in your neurology practice? |  |  |  |  |
|  |  |  |  |  |
| **Pathology** |  |  |  |  |
| 1. Understand hematological cytological interpretation |  |  |  | 3.5 |
| 2. Understand the technique to acquire CSF samples in small animals |  |  |  | 3.94 |
| 3. Understand the technique to acquire CSF samples in large animals |  |  |  | 3.54 |
| 4. Exhibit competence in CSF cytological interpretation in small animals |  |  |  | 3.8 |
| 5. Exhibit competence in CSF cytological interpretation in horses/ruminants/food animals |  |  | 3.33 |  |
| 6. Exhibit competence in CSF sample examination (protein content, cell counting) |  |  |  | 3.68 |
| 7. Understand basic CNS pathological interpretation |  |  |  | 3.75 |
| 8. Understand basic PNS pathological interpretation |  |  |  | 3.66 |
| 9. Understand microscopic pathological features of specific small animal diseases |  |  |  | 3.52 |
| 10. Understand microscopic pathological features of specific horse diseases |  |  | 3.16 |  |
| 11. Understand microscopic pathological features of specific ruminant / food animal disease |  |  | 3.09 |  |
| 12. Understand Infectious disease testing techniques (PCR / Western blot / Serology) |  |  | 3.21 |  |
| 13. Understand Infectious disease testing interpretation |  |  |  | 3.59 |
| 14. Exhibit competence in bone marrow cytological interpretation |  |  | 2.69 |  |
| 15. Exhibit competence in brain biopsy cytological interpretation |  |  | 3.01 |  |
| * What other aspects of pathology (besides those listed above) do you think should form part of the ECVN Residency Training? |  |  |  |  |
| * Which Clinical Pathology procedures do you perform in your neurology practice or do you have performed through a laboratory? |  |  |  |  |
|  |  |  |  |  |
| **Competencies of Academia** |  |  |  |  |
| 1. In teaching for undergraduates |  |  |  | 3.67 |
| 2. In teaching for postgraduates |  |  |  | 3.68 |
| 3. In veterinary education (Didactics) |  |  |  | 3.58 |
| 4. In study design |  |  |  | 3.49 |
| 5. In statistics |  |  | 3 |  |
| 6. In principles of evidence based medicine |  |  |  | 3.55 |
| 7. In good clinical practice |  |  |  | 3.78 |
| 8. In laboratorium |  |  | 3.12 |  |
| 9. In epidemiology |  |  | 3.06 |  |
| * What other aspects of competencies of academia (besides those listed above) do you think should form part of the ECVN Residency Training? |  |  |  |  |
| *What additional training as part of the ECVN Residency Programme do you think differentiates an ECVN Diplomate from an Advanced Veterinary Practitioner, specifically with regard to subsequently allowing the one to act as an ECVN Residency Supervisor, but not the other? |  |  |  |  |
|  |  |  |  |  |

* Free text questions
