# Supplementary material for: Development of learning objectives for neurology in a veterinary curriculum: Part II: Postgraduates
Source: BMC Vet Res. 2015 Jan 27;11:10. doi: 10.1186/s12917-014-0314-4 (PMC4323235; doi:10.1186/s12917-014-0314-4)
Supplement: Additional file 2: — Difference between ECVN Diplomates and advanced practitioners. [file 12917_2014_314_MOESM2_ESM.doc]

**Development of learning objectives for neurology in a veterinary curriculum. Part II: Postgraduates**

**Additional file 2**Difference between ECVN Diplomates and advanced practitioners

|  | |  | | |
| --- | --- | --- | --- | --- |
| **Are there any other skills/competencies which can differentiate a general practitioner and an advanced practitioner from a Diplomate?** | | **For general practitioners** | **For advanced practitioners** | **For diplomates** |
| 1. A high level of competency through teaching, research or practice in the specialty. | |  |  |  |
|  | True |  |  |  |
|  | False |  |  |  |
| 2. Acquisition and understanding of a substantial body of knowledge which is at the forefront of the area of veterinary professional practice. | |  |  |  |
|  | True |  |  |  |
|  | False |  |  |  |
| 3. The ability to apply high level knowledge and skills at the forefront of the specialist area to their own professional work. | |  |  |  |
|  | True |  |  |  |
|  | False |  |  |  |
| 4. A high level of clinical expertise in their specialty area including the ability to deal with non-routine and complex cases. | |  |  |  |
|  | True |  |  |  |
|  | False |  |  |  |
| 5. A detailed understanding of applicable techniques for research and clinical enquiry, including ability to design and implement a project for the generation of new knowledge, clinical methodologies and techniques at the forefront of the professional area. | |  |  |  |
|  | True |  |  |  |
|  | False |  |  |  |
| 6. Make informed judgements on complex issues in their specialist field, often in the absence of complete data, and be able to communicate their ideas and conclusions clearly and effectively to specialist and non-specialist audiences, including clients. | |  |  |  |
|  | True |  |  |  |
|  | False |  |  |  |
| 7. Continue to undertake research and/or clinical studies in their field at an advanced level, contributing substantially to the development of new knowledge, techniques, ideas or approaches in the specialty. | |  |  |  |
|  | True |  |  |  |
|  | False |  |  |  |
| * What part of the Residency Training and subsequent ECVN College organisation do you think differentiates an ECVN Diploma holder from an advanced practitioner who provides a neurology referral service? | |  |  |  |

* Free text questions
